# The Actin Binding Protein Plastin-3 Is Involved in the Pathogenesis of Acute Myeloid Leukemia

**DOI:** 10.3390/cancers11111663

**Published:** 2019-10-26

**Authors:** Arne Velthaus, Kerstin Cornils, Jan K. Hennigs, Saskia Grüb, Hauke Stamm, Daniel Wicklein, Carsten Bokemeyer, Michael Heuser, Sabine Windhorst, Walter Fiedler, Jasmin Wellbrock

**Affiliations:** 1 Department of Oncology, Hematology and Bone Marrow Transplantation with Division of Pneumology, Hubertus Wald University Cancer Center, University Medical Center Hamburg-Eppendorf, 20246 Hamburg, Germany; arne.velthaus@gmx.de (A.V.); j.hennigs@uke.de (J.K.H.); stamm.hauke@gmail.com (H.S.); cbokemeyer@uke.de (C.B.); fiedler@uke.de (W.F.); 2 Department of Pediatric Hematology and Oncology, Division of Pediatric Stem Cell Transplantation and Immunology, University Medical Center Hamburg-Eppendorf, 20246 Hamburg, Germany; k.cornils@uke.de; 3 Research Institute Children’s Cancer Center Hamburg, 20246 Hamburg, Germany; 4 Center for Experimental Medicine, Institute of Biochemistry and Signal Transduction, University Medical Center Hamburg-Eppendorf, 20246 Hamburg, Germany; s.grueb@uke.de (S.G.); s.windhorst@uke.de (S.W.); 5 Department of Anatomy and Experimental Morphology, University Cancer Center, University Medical Center Hamburg-Eppendorf, 20246 Hamburg, Germany; d.wicklein@uke.de; 6 Hematology, Hemostasis, Oncology and Stem Cell Transplantation, Hannover Medical School, 20246 Hannover, Germany

**Keywords:** Acute myeloid leukemia, Plastin-3, prognostic marker

## Abstract

Leukemia-initiating cells reside within the bone marrow in specialized niches where they undergo complex interactions with their surrounding stromal cells. We have identified the actin-binding protein Plastin-3 (PLS3) as potential player within the leukemic bone marrow niche and investigated its functional role in acute myeloid leukemia. High expression of PLS3 was associated with a poor overall and event-free survival for AML patients. These findings were supported by functional in vitro and in vivo experiments. AML cells with a PLS3 knockdown showed significantly reduced colony numbers in vitro while the PLS3 overexpression variants resulted in significantly enhanced colony numbers compared to their respective controls. Furthermore, the survival of NSG mice transplanted with the PLS3 knockdown cells showed a significantly prolonged survival in comparison to mice transplanted with the control AML cells. Further studies should focus on the underlying leukemia-promoting mechanisms and investigate PLS3 as therapeutic target.

## 1. Introduction

Acute myeloid leukemia (AML) has still a dismal prognosis due to persistence of minimal residual disease (MRD) which results in high relapse rates. Chemotherapy-resistant and therefore non-targetable leukemia-initiating cells are the cause for MRD [1,2]. The leukemia-initiating cells reside within the bone marrow (BM) in specialized niches where they undergo complex interactions with their surrounding stromal cells [3]. This microenvironment offers protection against external influences [4]. One of the major goals of modern therapeutic approaches is thus to elucidate the complex signaling within the leukemic bone marrow niche as it might represent a rich source of novel therapeutic targets.

In the current study, we investigated the impact of Plastin-3 (PLS3) in acute myeloid leukemia as we had identified PLS3 as potential player within the leukemic bone marrow niche through gene expression analysis of AML and endothelial cell co-cultures.

Plastins, which are also called fimbrins, belong to a subclass of actin-binding proteins known as actin bundling proteins that can cross-link actin filaments into higher-order assemblies like bundles. In mammals, three isoforms have been characterized: Plastin-1 (PLS1 or I-Plastin) is specifically expressed in the small intestine, colon and kidney. Plastin-2 (PLS2 or L-Plastin) is predominantly expressed in hematopoietic cells, while Plastin 3 (PLS3 or T-plastin) is described to be expressed in cells of non-hematopoietic origin [5,6]. In addition, to its actin-bundling capacity, PLS3 may also control actin turnover, stabilization and assembly [7].

## 2. Results

### 2.1. The Actin Binding Protein PLS3 Is Expressed in Primary AML Cells

**Figure 1:**
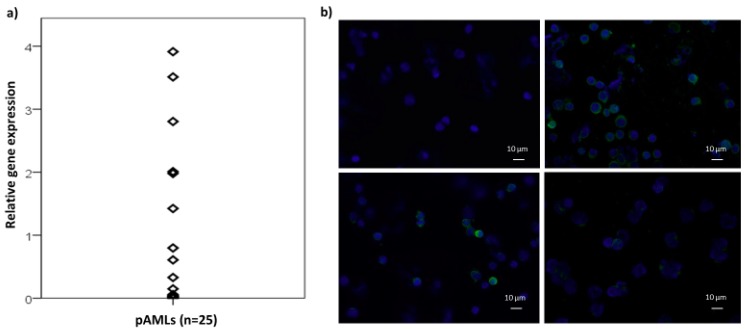
Expression of PLS3 in primary AML samples. (**a**) The mRNA expression of primary AML samples was analyzed by RT-qPCR and normalized to GAPDH. The relative expression is shown in comparison to Kasumi-1 cells (expression value of 1). (**b**) PLS3 protein expression in primary AML samples was analyzed by immunofluorescence (green signals). The panel upper left shows the negative control without primary antibody while the lower left and right panels show the PLS3 staining of three primary AML samples of varying PLS3 expression intensity, respectively.

In order to identify genes being implicated in the interaction of acute myeloid leukemia (AML) cells and stromal cells, we performed co-cultures of primary AML cells with primary endothelial cells and screened for genes that were up- or down-regulated under the co-culture conditions by microarray analysis (Human Gene 2.0 ST Array, Affymetrix, Thermo Fisher Scientific, Waltham, MA, USA). Co-cultures of CD34 positive hematopoietic progenitor cells from healthy donors were analyzed in parallel. Our aim was the identification of genes that were exclusively expressed in the leukemic cells. The actin binding protein PLS3 (Plastin-3) was identified to be up-regulated in the AML cells co-cultured with endothelial cells (9.8 +/− 5.0 fold up-regulation) while no significant alteration was observed in the co-culture of CD34 positive hematopoietic progenitor cells and endothelial cells (1.6 +/− 1.3 fold up-regulation; Supplementary Table S1). Further RT-qPCR analysis revealed an endogenous PLS3 expression in about 50% of BM samples from AML patients (*n* = 25; Figure 1a). The PLS3 expression in primary AML cells was verified at the protein level by immunofluorescence (*n* = 9, Figure 1b). In contrast, mRNA expression of PLS3 was only detected in 2 of 12 AML cell lines analyzed with Kasumi-1 showing strong and THP-1 showing only very weak expression. Therefore, we concentrated our functional analysis of PLS3 on the AML cell line Kasumi-1.

### 2.2. Functional Analysis of PSL3 by Knockdown or Overexpression in Kasumi-1 Cells

To study the functional effect of PLS3 on the cell biology, we performed a shRNA-mediated knockdown as well as overexpression of PLS3 in Kasumi-1 cells by lentiviral transduction. For the knockdown, we used two different PLS3-targeting shRNA constructs (PLS3-shRNA1 and PLS3-shRNA2) which resulted both in an efficient PLS3 mRNA knockdown of 80% to 90% (supplemental Figure S1). For the PLS3 overexpression, we cloned a vector with a N-terminal (PLS3-GFP) and a C-terminal (GFP-PLS3) PLS3 and GFP fusion construct, respectively. The relative mRNA overexpression of the PLS3-GFP- and GFP-PLS3-transduced Kasumi-1 cells was 18-fold and 35-fold, respectively (supplemental Figure S1). Differences between the PLS3 knockdown and overexpression cells in comparison to their respective controls were studied with different functional in vitro assays.

**Figure 2:**
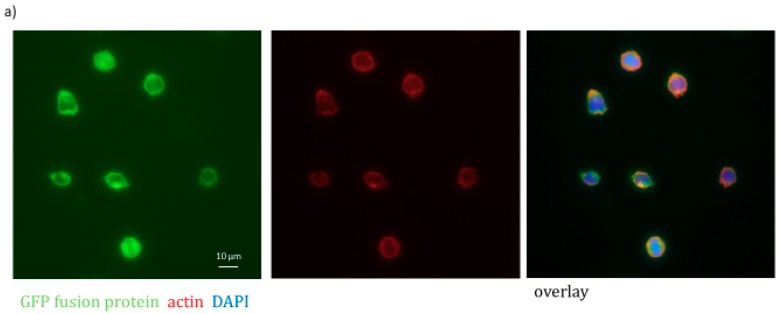

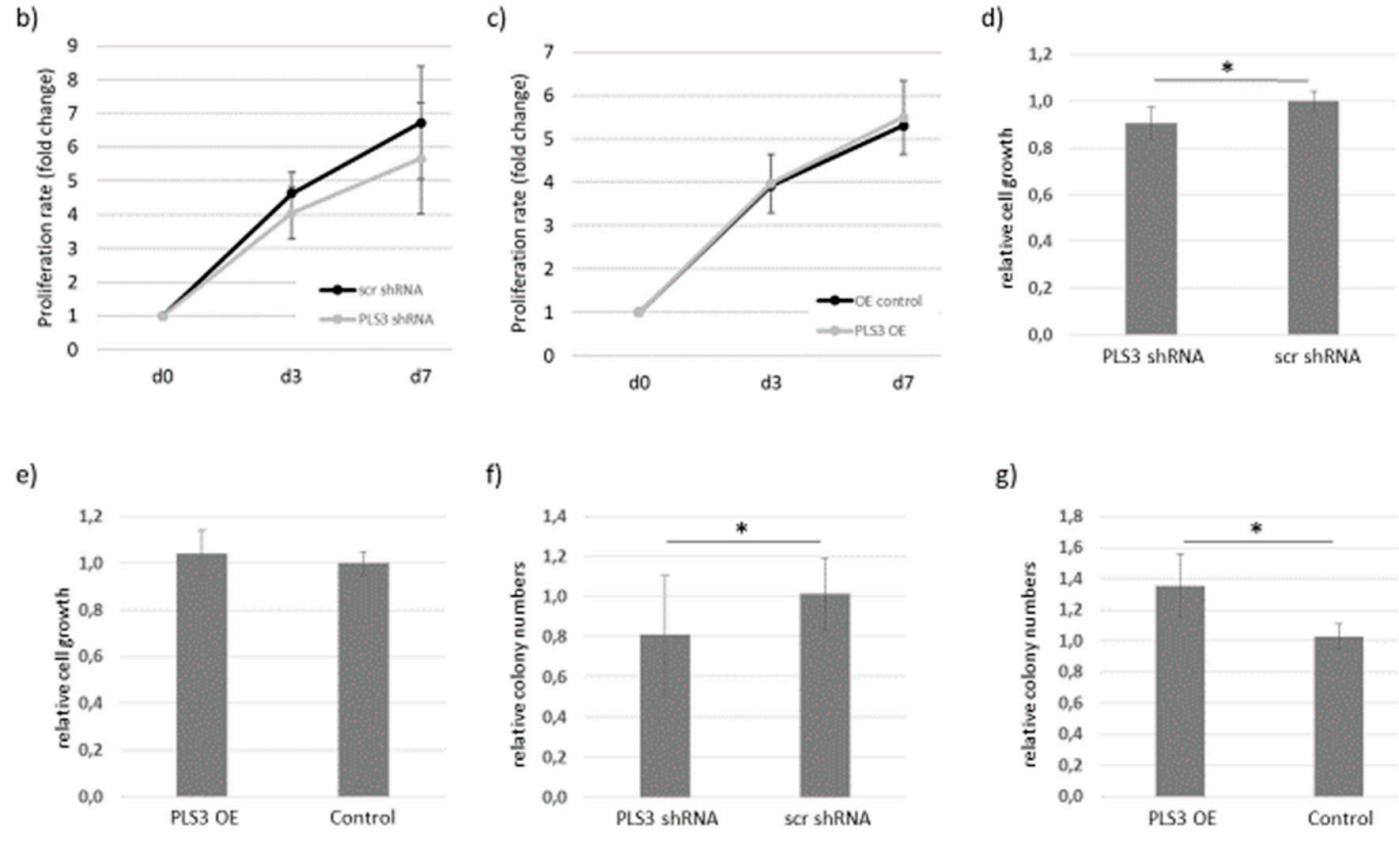
Functional in vitro assays with PLS3 knockdown and overexpression Kasumi-1 cells. (**a**) GFP PLS3 overexpression cells were used to study the co-localization of PLS3 and F-actin. F-actin was labeled with Alex-fluor568-conjugated phalloidin (red). (**b**–**e**) Proliferation of PLS3-knockdown (**b**,**d**) and PLS3-overexpression (**c**,**e**) Kasumi-1 cells were analyzed in proliferation assays over 7 days. (**b**,**c**) Show a growth curve over 7 days, (**d**,**e**) show the bar graphs of the relative cell growth on day 7. (**f**,**g**) The colony formation capacity of PLS3-knockdown (**f**) and PLS3-overexpression (**g**) Kasumi-1 cells were analyzed in colony formation assays over 7 days. * *p* < 0.05.

As PLS3 is an actin-binding protein, we first investigated co-localization of PLS3 and F-actin in Kasumi-1 overexpressing cells. As shown in Figure 2a, a clear co-localization of PLS3 and F-actin could be observed. Next, we studied whether PLS3 had an influence on the growth of Kasumi-1 cells in vitro. We could detect a slightly decreased proliferation rate when PLS3 was knocked down in Kasumi-1 cells but no significant increase in the PLS3 overexpression variants in proliferation assays (Figure 2b–e). AML is believed to be hierarchical with leukemia-initiating cells at the top that generate the pool of all leukemic progeny. The in vitro colony formation capacity mirrors the ability to give rise to leukemic progeny. Therefore, we analyzed whether PLS3 has an impact on the colony formation capacity of AML cells. Kasumi-1 cells transduced with PLS3-specific shRNAs or PLS3 overexpression constructs were seeded in methylcellulose-based semi-solid medium, and the number of colonies was counted after 7 to 10 days. We found that Kasumi-1 cells with a PLS3 knockdown showed significantly reduced colony formation capacity in comparison to cells transduced with a control vector (*p* < 0.0001, Figure 2f). In line with these data, we found significantly increased colony numbers in the PLS3 overexpressing Kasumi-1 cells (*p* < 0.001; Figure 2g).

In order to get more insight into the signaling cascades PLS3 might be involved in leukemia, we performed RNA sequencing of Kasumi-1 PLS3-sh1 and PLS3-sh2 knockdown vs. control cells. Although the knockdown reached nearly 90% for both shRNA variants, to our surprise the overall gene dysregulations in the knockdown cells were only modest. Nevertheless, with an adjusted *p*-value of *p* < 0.1, we could detect ten genes and two non-coding RNAs that were significantly dysregulated in both PLS3 knockdown variants in comparison to their control cells. Dysregulated genes or non-coding RNAs included the fasciculation and elongation protein zeta (FEZ1), multimerin 1 (MMRN1) or RGMB antisense RNA 1 (RGMB-AS1; see Supplementary Table S2 for complete list). Using publicly available gene expression data of a large cohort of AML patients (GEO accession number GSE6891), we analyzed whether the gene expression level of PLS3 was correlated to the gene expression levels of the identified genes by performing a Pearson correlation [8]. Significant correlations could be found for PLS3 and ENPP3 (for one of two probesets, *r* = 0.279, *p* < 0.001), KIAA1751 (for three of four probesets, *r* = 0.182, *r* = 0.294, *r* = 0.232, respectively, *p* < 0.001 for all), MMRN1 (*r* = 0.157, *p* < 0.001), POU4F3 (*r* = 0.294, *p* < 0.001), RSPH10B (*r* = 0.310, *p* < 0.001) and RGMB-AS1 (*r* = 0.228, *p* < 0.001).

### 2.3. Knockdown of PLS3 Significantly Improves Survival In Vivo

**Figure 3:**
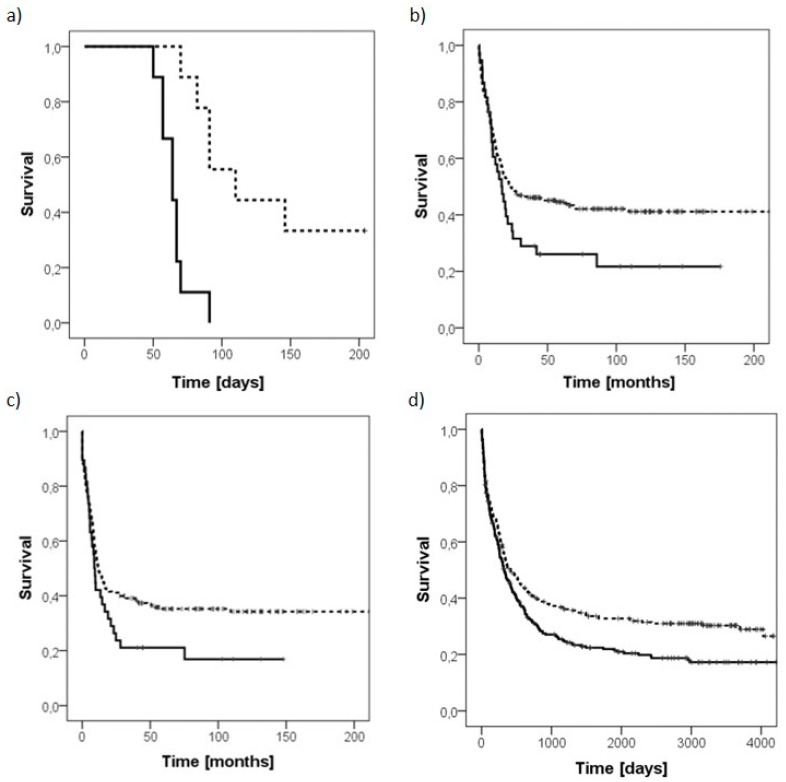
High PLS3 expression is associated with a poor prognosis. (**a**) Kaplan–Meier survival curve of NSG mice transplanted with PLS3-knockdown (dashed line) or control Kasumi-1 cells (continuous line; *n* = 9 mice per group; *p* < 0.001). (**b**,**c**) A clear difference for high PLS3 expression (continuous line) versus low PLS3 expression (dashed line) on overall (OS) and event-free (EFS) survival was observed in a publicly available AML patient cohort (*n* = 293; *p* = 0.062 for OS and *p* = 0.067 for EFS). (**d**) High PLS3 expression levels (continuous line) were associated with a poor overall survival in comparison to low PLS3 expression levels (dashed line) in a second independent AML patient cohort (*n* = 553; *p* = 0.009).

To investigate the role of PLS3 in vivo, NSG mice were transplanted with the PLS3 knockdown Kasumi-1 cells. Compared to mice transplanted with Kasumi-1 control cells transduced with a vector carrying a scrambled shRNA, the PLS3 knockdown mice survived significantly longer (median survival time 64 vs. 110 days, respectively; *p* < 0.001; *n* = 9 mice per group; Figure 3a). Moreover, three animals of the PLS3 knockdown group did not show any signs of leukemia 200 days after transplantation of Kasumi-1 cells. Therefore, the experiment was terminated, and the three animals were sacrificed and analyzed for leukemic infiltration by flow cytometry. Remarkably, no tumors or leukemia infiltration in the peripheral blood, bone marrow, spleen or liver could be detected.

### 2.4. PLS3 Represents a Negative Prognostic Marker in AML

Finally, we investigated whether expression of PLS3 was associated with AML patients’ outcome using published microarray-based gene expression data (GEO accession number GSE6891) [8]. Clinical data of 290 AML patients were available. Based on the mean gene expression value, the patient cohort was divided into high vs low PLS3 expressors. The overall survival and event-free survival was analyzed in a multivariate Cox proportional hazards model including PLS3 gene expression and the baseline parameters age, karyotype and FLT3 mutational status. After a stepwise removal of insignificant terms, the patient’s age and a high PLS3 expression remained as independent prognostic markers for the overall survival (for PLS3: *p* = 0.028, HR 1.58 (CI 1.05–2.37) and for age: *p* = 0.036, HR 1.01 (CI 1.00–1.03)). Regarding the EFS, none of the analyzed factors remained significant in the multivariate analysis but PLS3 had the strongest impact and was borderline significant (*p* = 0.071, HR 1.39 (CI 0.97–2.09)). There was no significant difference regarding the distribution of high PLS3 expressors in FAB subtypes M1, M2, M4 or M5 although the frequency was slightly lower in subtypes M2 and M4 (Table 1). Due to the low number of 20 cases in total, FAB subtypes M0, M3, M6 and M7 were excluded from the subgroup analysis.

The negative prognostic impact of PLS3 could be verified in a second publicly available AML patient cohort where high PLS3 expression was associated with a poor overall survival (*p* = 0.009; *n* = 553 patients, GEO accession number GSE37642) [9]. The respective Kaplan–Meier survival curves are shown in Figure 3b–d.

## 3. Discussion

In the current study, the actin binding protein PLS3 was identified as potential player in acute myeloid leukemia. We found that high expression of PLS3 was associated with a poor overall and event-free survival for AML patients in two independent patient cohorts. These data are supported by our in vitro and in vivo experiments. Kasumi-1 cells with a shRNA-mediated PLS3 knockdown showed significantly reduced colony numbers in vitro while PLS3 overexpression resulted in significantly enhanced colony numbers compared to their respective controls. The clonogenicity of cancer cells mirrors their stem cell potential, which represents an important characteristic for the engraftment and establishment of cancer cells in vivo. In line with this consideration, the survival of NSG mice transplanted with the PLS3 knockdown cells was significantly prolonged in comparison to mice transplanted with the control Kasumi-1 cells. More importantly, three animals of the knockdown group did never develop a leukemia confirming our hypothesis that PLS3 is important for the leukemic engraftment.

The involvement of PLS3 in cancer biology was described in a number of studies. For example, in patients with Sezary syndrome, a leukemic and erythrodermic variant of cutaneous T cell lymphomas, upregulation of PLS3 has been observed which is in part due to the hypomethylation of the PLS3 promoter [10,11]. Furthermore, PLS3 was identified as negative prognostic marker in gastric cancer as patients with high PLS3 expression levels had a higher incidence of advanced tumor stage, cancer differentiation, tumor invasion depth, distant metastases and a significantly poorer prognosis than the low expression group. In vitro, gastric cancer cell lines transduced with PLS3 siRNA exhibited reduced migration and invasiveness [12]. Szkandera et al. could show that a common gene variant in PLS3 represents an independent prognostic marker in female patients with stage II and stage III colon cancer as the occurrence of the polymorphism was associated with a decreased time to recurrence [13]. Moreover, PLS3 expression was discovered as potential marker for circulating tumor cells undergoing epithelial-mesenchymal transition in a variety of cancers. PLS3 expression on circulating tumor cells was described in metastatic colorectal cancer patients. Furthermore, multivariate analysis showed that the occurrence of PLS3-positive circulating tumor cells was independently associated with the patients’ prognosis [14]. In another study, PLS3 expression on circulating tumor cells could be linked to lymph node metastasis [15]. Ueo and colleagues observed PLS3 expression in circulating tumor cells of patients with breast cancer. Furthermore, the PLS3-positive patients showed significantly poorer overall and disease-free survival than PLS3-negative patients. Subset analysis revealed that this prognostic biomarker was relevant in patients with stage I-III cancer, particularly in patients with luminal-type and triple-negative-type tumors [16]. These observations, namely, the association with a poor outcome and expression in circulating tumor cells, indicate that PLS3 might be involved in the process of metastasis. This finding is of special interest as leukemic engraftment and maintenance of leukemia-initiating cells within their stem cell niche has presumably parallels to the process of metastasis of solid tumors.

Additionally, PLS3 was also associated with therapy resistance. For example, Ma and colleagues could show that PLS3 silencing in triple-negative breast cancer cells increased the sensitivity to paclitaxel via the p38 MAPK signaling cascade [17]. Furthermore, several studies indicate that PLS3 might be involved in the cellular response to DNA-damaging agents such as cisplatin. Cisplatin is an anticancer agent binding to DNA and interfering with DNA repair. Cisplatin-resistant cancer cell lines including bladder, prostate or head and neck cancer showed higher PLS3 levels compared to their parental cell lines. Furthermore, the reduction of PLS3 expression using antisense RNA in a bladder cancer cell line was associated with increased sensitivity to cisplatin [18]. Moreover, T-plastin was also upregulated in UV radiation-resistant cells [19]. In Chinese hamster ovary (CHO) cells with an X-radiation-induced G2 arrest, increased PLS3 expression was observed. The G2 arrest levels decreased upon downregulation of PLS3 indicating a correlation between PLS3 and G2/M cell-cycle control [20]. Resistance to chemotherapy might contribute to the poor outcome of the PLS3 high expressors in AML.

Mutations in PLS3 cause X-linked primary osteoporosis in men and furthermore, the occurrence of osteoporosis in elderly women after the menopause is associated with a rare single nucleotide polymorphism in PLS3. In line with these data, PLS3 knockout mice exhibit osteoporosis while PLS3 overexpressing mice show thickening of cortical bone due to disturbed osteoclast function. High PLS3 levels were associated with upregulation of the NFκB subunit p65 [21]. Interestingly, NFκB (comprised of subunits p65 and p50) is a potent transcriptional activator of the cMYC promoter and additionally inhibits the cMYC protein degradation [22,23]. The MYC oncogene contributes to the genesis of many human cancers and cMYC is an important prognostic factor in AML [24,25]. PLS3 might mediate its leukemia-promoting effects in our study at least partly through activation of cMYC.

As PLS3’s functional role is not completely understood, we performed RNA sequencing analysis of the PLS3-knockdown cells. Although we reached a knockdown of more than 90%, only very few overlapping genes were found in the two different shRNAs targeting PLS3. The fasciculation and elongation zeta-1 protein (FEZ1) was one of those genes. FEZ1 is supposed to be a tumor suppressor in several cancer entities including bladder, breast or lung cancer [26,27,28]. But interestingly in leukemia, dysregulated FEZ1 might have opposing effects. Overexpression of FEZ1 in HEK293 and HeLa cells resulted in multi-lobulated nuclei as often observed in human leukemia cells [29]. Furthermore, it was shown that FEZ1 interacts with the retinoic acid receptor (RAR) thereby regulating the transcription of the HOXB4 gene [30]. HOXB4 has been implicated as a tumor-related gene in many cancer entities, including leukemia where it is also related to poor prognosis and chemoresistance [31,32,33]. MMRN1 which is a factor V/Va binding protein that might also be involved in extracellular matrix processes was also identified by RNA sequencin [34]. No functional relation to cancer biology is known, but MMRN1 was identified as negative prognostic marker in pediatric AML [35]. Additionally, the non-coding RNA RGMB-AS1 was downregulated in the PLS3-knockdown cells. RGMB-AS1’s role in cancer is controversial. On the one hand, high RGMB-AS1 levels were associated with advanced clinical features in laryngeal squamous cell carcinoma and lung adenocarcinoma [36,37] while on the other hand it was identified as favorable prognostic marker in hepatocellular carcinoma [38]. Whether and how PLS3 is associated with one of the dysregulated genes or non-coding RNAs that have been identified by RNA sequencing should be investigated in further studies.

Taken together, we could identify the actin binding protein PLS3 as potential player for the establishment and maintenance of acute myeloid leukemia. Further studies should focus on the underlying leukemia-promoting molecular mechanisms and PLS3 should be investigated as therapeutic target.

## 4. Materials and Methods

### 4.1. Patients and Samples

Primary AML cells for in vitro experiments were obtained after patient’s informed consent and approval of the study by the ethics committee (PV3469, Ethik-Kommission der Ärztekammer Hamburg). Cells were isolated from bone marrow using density gradient centrifugation.

Furthermore, we analyzed two independent AML patient cohorts of whom microarray-based gene expression data was published by Verhaak et al. (cohort A, *n* = 290 patients, data accessible at NCBI GEO database, accession GSE6891) and Li et al. (cohort B, *n* = 553 patients, data accessible at NCBI GEO database, accession GSE37642), respectively [8,9]. For cohort A, patients were derived from a clinical study by Löwenberg et al. [39]. Patients aged between 15 and 60 years with newly diagnosed AML (APL excluded) received 3 cycles of standard chemotherapy. In a 2 × 2 factorial design patients were randomized to granulocyte-macrophage colony-stimulating factor either during chemotherapy only, after chemotherapy until recovery of blood counts, during both periods or none. Leukemia-specific outcome was independent of randomization. For cohort B, patients were enrolled into the German AMLCG 1999 trial. Patients received intensive standard induction chemotherapy followed by either autologous stem cell transplantation or consolidation therapy plus 3 years of maintenance chemotherapy.

### 4.2. Cell Culture

The human AML cell line Kasumi-1 was cultured in RPMI-1640 medium (Gibco) supplemented with 20% fetal bovine serum (FBS, Biochrom GmbH, Berlin, Germany). HEK293T cells were cultured in DMEM medium (Gibco, Thermo Fisher Scientific, Waltham, MA, USA) supplemented with 10% FBS. All cells were maintained in a humidified incubator with 5% CO_2_ at 37 °C.

### 4.3. Reverse Transcription Quantitative PCR Analysis

Total RNA was extracted using the innuSPEED Tissue RNA Kit (Analytik Jena, Jena, Germany) and transcribed into cDNA using the PrimeScript RT Master Mix (Clontech, Madison, WI, USA). Exon-spanning primers for PLS3 and glyceraldehyde 3-phosphate dehydrogenase (GAPDH) were designed with Primer 3 software (Whitehead Institute for Biomedical Research, Boston, MA, USA). PLS and GAPDH primers were as follows: PLS3 forward 5′-atttgtgctctgggtggaac-3′, PLS3 reverse 5′ acaggtcatcggtgttaggg-3′, GAPDH forward 5′ gtcagtggtggacctgacct 3′, GAPDH reverse 5′ tgctgtagccaaattcgttg-3′. Reverse transcription quantitative PCR analysis was carried out on the LightCycler 96 (Roche, Basel, Switzerland) using the SYBR Premix Ex Taq 2 Kit (Takara, Tokyo, Japan) over 40 PCR cycles. PCR efficiencies were calculated using a standard curve obtained from log dilutions of a positive cDNA sample. Samples were analyzed in triplicates and averaged. The relative mRNA expression of PLS3 was calculated using a method introduced by Pfaffl [40]. Expression of GAPDH served as reference gene.

### 4.4. Immunofluorescence Staining

The whole leukemic mononuclear cell fraction of AML bone marrows was spun onto glass slides using the Rotofix 32A centrifuge (Hettich Zentrifugen, Tuttlingen, Germany). Slides were fixed in 4% paraformaldehyde/PBS for 10 min, permeabilized in 0.1% Triton-X 100 for 5 min and then blocked for 60 min with 10% normal donkey serum. The primary PLS3 antibody (dilution 1:500, #PA5-27883; Invitrogen, Carlsbad, CA, USA) was incubated overnight followed by incubation with a secondary AF488-conjugated donkey anti-rabbit IgG (1:400, #A21206, Invitrogen) for 1 h and staining with DAPI.

### 4.5. Lentiviral Transduction of Kasumi-1 Cells with PLS3-Specific shRNA or a PLS3 Overexpression Construct

pLKO.1-puro vector encoding PLS3-targeting (sh1 sequence 5′-CCGGGCTCAGAACTTAGACGGGATTCTCGAGAATCCCGTCTAAGTTCTGAGCTTTTTG-3′ and sh2 sequence 5′-CCGGGCTGAGAGTATGCTTCAACAACTCGAGTTGTTGAAGCATACTCTCAGCTTTTTG-3′) or scrambled shRNA (negative control) were purchased from Sigma-Aldrich (Taufkirchen, Germany). The shRNA sequences were cloned into the LeGO vector system (a 3rd generation HIV1-derived lentiviral vector) as they carry fluorescent marker proteins allowing easy testing of transduction efficiencies (www.LentiGO-Vectors.de) and genes for antibiotic resistance facilitating the selection of transduced cells. For the overexpression variants, a LeGO vector carrying the PLS3 coding sequence upstream or downstream of the GFP insert was constructed which resulted in a N-terminal (PLS3-GFP) and a C terminal (GFP-PLS3) PLS3 and GFP fusion construct, respectively. The GFP vector served as negative control.

HEK293T cells were used for the production of lentiviral particles that were harvested 24 h after transfection and added to Kasumi-1 cells. Transduced cells were selected by addition of puromycin (2 μg/mL; Sigma-Aldrich; for sh1-transduced and PLS3-GFP and GFP-PLS3 overexpression variants) or zeocin (2 μg/mL; Sigma-Aldrich; for sh2-transduced cells), respectively, for 4 days prior to functional assays. The knock-down and over-expression efficiency was analyzed in quantitative PCR analysis on day 4 of puromycin or zeocin selection. All work with lentiviral particles was done in a S2 facility after approval according to German law.

### 4.6. Staining of F-Actin

AML cells grown on poly-L-lysin coated chamber slides (Ibidi) were fixed with 4% paraformaldehyd/PBS for 10 min 37 °C. After washing the cells 3-times with PBS, they were incubated with Alexa-fluor 568-coupled phalloidin (Thermo Fisher Scientifics, Waltham, MA, USA), diluted 1:1000 in PBS, for 30 min at RT. Thereafter, the cells were washed again 3-times with PBS and stained with 4′,6-Diamidin-2-phenylindo (1:2000 in PBS) for 5 min at RT. After final washing, fluorescence was analyzed by the Keyence BZ 9000 microscope.

### 4.7. Proliferation Assays

Kasumi-1 cells with a PLS3 knockdown, PLS3 overexpression and their respective controls were seeded in triplicates in 24-well plates at a density of 0.3 × 10^6^ cells/mL. Cell numbers were determined on day 3 and day 7 using the cell viability analyzer Vi-Cell XR (Beckman Coulter, Brea, CA, USA).

### 4.8. Colony Formation Assay

The colony formation capacity of AML cells with the PLS3 knockdown or overexpression was compared to their respective controls. AML cells were seeded in Methocult (Methocult H4230, Stemcell Technologies) at a density of 250 cells/mL. The number of colonies was counted after 7 days using an inverted microscope (Axiovert 25, Carl Zeiss Microscopy GmbH, Jena, Germany).

### 4.9. Xenograft Model

1 × 10^6^ Kasumi-1 PLS3 knockdown or scrambled-shRNA cells were intravenously transplanted into female NSG (NOD.Cg-Prkdcscid Il2rgtm1Wjl/SzJ) mice, respectively (9 animals per group). Mice were sacrificed when showing clear symptoms of leukemia such as dramatic loss of weight, tumor weight, hypothermia or apathy. Three remaining mice in the control group showing no signs of leukemia, were sacrificed 200 days after transplantation and checked for leukemic infiltration in the peripheral blood, bone marrow, spleen and liver by flow cytometry using a human-specific CD45 antibody (Clone HI30, Biolegend, San Diego, CA, USA).

### 4.10. RNA Sequencing

Total RNA was extracted from lentivirally transduced Kasumi-1 cells with RNeasy Mini Kit (Qiagen, Hilden, Germany), and opposing strand-specific library preparation was performed with the NEBNext Ultra II RNA directional Kit (New England Biolabs, Hoechst, Germany) and single read sequencing was performed using a NextSeq^®^ 500 System (Illumina, San Diego, CA, USA) with a read length of 75 bp. Samples were demultiplexed (bcl2fastq2), quality controlled (FastQC) and groomed (FastQ groomer) using the Stanford Genetics Bioinformatics Service Center Galaxy platform (Stanford, CA, USA). Resulting reads were aligned to the human genome using the built-in hg19 reference genome utilizing STAR aligner. The aligned reads were transferred into SeqMonk 1.45 (Babraham Institue) and quantified using the built-in RNA quantification pipeline on merged transcripts of two different PLS3-shRNAs combined versus control shRNA. Differentially expressed genes (DEGs) were defined as significantly regulated merged transcripts by LIMMA analysis (FDR < 0.05, Benjamin–Hochberg correction).

### 4.11. Statistics

All statistical analyses were done with SPSS 21 (SPSS Inc., Chicago, IL, USA) and R 3.5.0 (R Foundation for Statistical Computing, Vienna, Austria) utilizing the BioConductor repository. Overall survival (OS) was defined as time from study inclusion to death. Event-free survival (EFS) was defined as time from study inclusion to any predefined event (first therapy failure, relapse or death). Kaplan–Meier survival curves were calculated for different categories and compared with log-rank tests. The mean PLS3 expression level was used to divide the cohort into low and high expressors. To identify those gene expressions with independent significant predictive power, gene expressions were entered simultaneously into the same multivariable Cox model, and a backwards selection was applied. Differences in proliferation or colony forming capacities were accessed by Mann–Whitney U tests. For all analyses, a *p*-value of *p* < 0.05 was considered statistically significant.

## 5. Conclusions

We could identify the actin binding protein PLS3 as potential player for the establishment and maintenance of acute myeloid leukemia. Further studies should focus on the underlying leukemia-promoting molecular mechanisms and PLS3 should be investigated as therapeutic target.

## Figures and Tables

**Table 1:** Distribution of low vs. high PLS3 expressors in different FAB subtypes.

FAB Subtype	PLS3 (%)		
	Low	High	Fisher’s Exact Test
M1 (*n* = 67)	81	19	*p* = 0.077
M2 (*n* = 64)	94	6	
M4 (*n* = 59)	93	7	
M5 (*n* = 62)	97	13	

## Supplementary Materials

The following are available online at https://www.mdpi.com/2072-6694/11/11/1663/s1, Figure S1. Knockdown and overexpression efficiency of PLS3 in Kasumi-1 cells. Table S1: Microarray data of PLS3 gene expression alteration in the co-culture condition; Table S2: Genes and non-coding RNAs down-regulated in the PLS3 knockdown cells.
