# Supplementary material for: The Actin Binding Protein Plastin-3 Is Involved in the Pathogenesis of Acute Myeloid Leukemia
Source: Cancers (Basel). 2019 Oct 26;11(11):1663. doi: 10.3390/cancers11111663 (PMC6895973; doi:10.3390/cancers11111663)
Supplement: Supplementary file 1 [file cancers-11-01663-s001.pdf]

*Supplementary Materials*

# The Actin Binding Protein Plastin-3 Is Involved in the Pathogenesis of Acute Myeloid Leukemia

**Arne Velthaus, Kerstin Cornils, Jan K. Hennigs, Saskia Grüb, Hauke Stamm, Daniel Wicklein, Carsten Bokemeyer, Michael Heuser, Sabine Windhorst, Walter Fiedler and Jasmin Wellbrock**

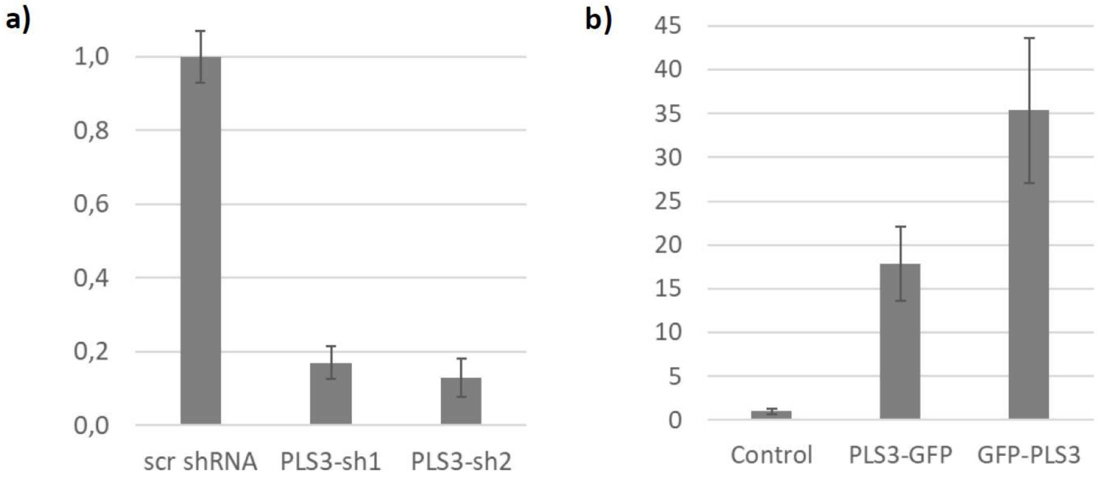


**Figure S1.** Knockdown and overexpression efficiency of PLS3 in Kasumi-1 cells.

**Table 1.** Microarray data of PLS3 gene expression alteration in the co-culture condition.

| Sample | Expression alteration in co-culture vs. mono-culture |
|---|---|
| **AML-1** | 6,00 |
| **AML-2** | 1,85 |
| **AML-3** | 2,05 |
| **CD34-1** | 1,14 |
| **CD34-2** | 0,42 |
| **CD34-3** | 0,44 |

**Table S2.** Genes and non-coding RNAs down-regulated in the PLS3 knockdown cells.

| *Gene* | *Fold Change* | *FDR* |
|---|---|---|
| *ENPP3* | 0.5 | <0.001 |
| *FEZ1* | 0.5 | <0.001 |
| *KIAA1751* | 0.5 | <0.001 |
| *MAP3K7CL* | 0.5 | <0.001 |
| *MMRN1* | 0.5 | <0.001 |
| *POU4F3* | 0.5 | <0.001 |
| *RGMB-AS1* | 0.5 | <0.001 |
| *RSPH10B* | 0.5 | <0.001 |
| *RSPH10B2* | 0.5 | <0.001 |
| *SEC14L5* | 0.25 | <0.001 |
| *SEPT3* | 0.5 | <0.001 |
| ***Non-coding RNA*** | | |
| *RGMB-AS1* | 0.5 | <0.001 |
| *SMARCA5-AS1* | 0.5 | <0.001 |

© 2019 by the authors. Licensee MDPI, Basel, Switzerland. This article is an open access article distributed under the terms and conditions of the Creative Commons Attribution (CC BY) license (http://creativecommons.org/licenses/by/4.0/).
